# The Management of Root Perforation: A Review of the Literature

**DOI:** 10.7759/cureus.72296

**Published:** 2024-10-24

**Authors:** Maram M Alshehri, Bushra F Alhawsawi, Atheer Alghamdi, Salem O Aldobaikhi, Maha H Alanazi, Fahad A Alahmad

**Affiliations:** 1 College of Dentistry, King Saud Bin Abdulaziz University for Health Sciences, Riyadh, SAU; 2 Restorative Department, Riyadh Elm University, Riyadh, SAU; 3 College of Dentistry, King Saud University, Riyadh, SAU; 4 Dentistry, Jubail General Hospital, Jubail, SAU; 5 Department of Dentistry, Armed Forces Hospital, Jizan, SAU

**Keywords:** apical perforation, bioceramic materials, coronal perforation, endodontic perforation, iatrogenic perforation, mineral trioxide aggregate, pathological perforation

## Abstract

Root canal perforation is defined as an opening or communication of the root to the outer oral tissue that occurs either accidentally during root canal therapy or due to unknown pathological causes, which generally result in complications in patients due to the passage of microorganisms to the surrounding tissues. These complications may necessitate extraction of the affected tooth. In this literature review, 42 articles were reviewed initially; 33 of them were included in the final analysis based on exclusion and inclusion criteria. Root perforation may occur due to caries, external and internal resorption, trauma, as well as iatrogenic causes. Many studies have suggested that perforation can be identified by radiographs, unexpected bleeding and pain during instrumentation, blood on paper points, electronic apex locator, and dental operating microscope.

Several materials have been used in endodontic perforation repairs, such as indium foil, super 2-ethoxybenzoic acid (super EBA), as well as bioceramic materials like mineral trioxide aggregate (MTA) and biodentine. These materials must achieve biocompatibility, bacteriostatic ability, and radio-opacity. Many studies have reported the superior properties of MTA in comparison with other materials. Based on our literature review, management of root perforation should be performed based on multiple significant factors. Clinicians must possess a comprehensive understanding of tooth anatomy to prevent perforation occurrence. Furthermore, clinical management and prognosis of root perforation have been enhanced by the availability of advanced materials and sealing techniques.

## Introduction and background

Root canal perforation is defined as an opening in the root; additionally, according to the American Association of Endodontists Glossary of Endodontic Terms, it refers to mechanical or pathological connections between the root canal system and the external tooth surface [1,2]. Root perforation constitutes an undesirable complication in endodontic treatment, compromising the structural integrity of the root, and leading to the further destruction of adjacent periodontal tissues [3]. This perforation can occur either accidentally during endodontic therapy or due to other causes such as iatrogenic reasons, the resorptive process, or dental caries [4].

Root perforation can occur during root canal treatment, such as in the preparation of access cavity and post-space preparation. In contrast, perforation that occurs as a result of the extension of internal resorption into the periradicular tissues falls under the resorptive process [3]. Kvinnsland et al. have reported that 53% of iatrogenic perforations occur during post-space preparation in prosthodontic treatments, whereas the remaining 47% of cases are caused by routine endodontic procedures [5]. All types of perforation generally result in complications in the patients due to the passage of microorganisms to the surrounding tissues [1]. Complications from root perforation may necessitate the extraction of the affected tooth. In one study about the causes of extraction of endodontically treated teeth, 4.2% of teeth were extracted due to iatrogenic perforation and stripping [2,6].

The introduction of new and advanced materials and techniques in endodontic practice has enabled dentists to attempt a more conservative approach to repair perforation with promising results [3,7]. The prognosis of an endodontic perforation depends on the size and location of the defect, as well as prompt sealing of the perforation area with biocompatible material. A variety of materials, such as zinc oxide eugenol (ZOE), amalgam, Cavit, composite resin, glass-ionomer (GIC), and mineral trioxide aggregate (MTA), have been suggested for perforation sealing. Until now, MTA, which was developed at Loma Linda University in the 1990s as a root-end filling material, has been considered a superior material for root perforation repair [3]. The purpose of this paper is to review and analyze the etiology, diagnostic aids, and handling materials and techniques, as well as the prognosis of root perforation.

The article has been previously presented as an abstract at the 7th Health Professions Conference at KSAU-HS.

## Review

Methods

We conducted a comprehensive electronic search across the Saudi Digital Library, Google Scholar, and Pubmed by using the following keywords: "mineral trioxide aggregate, Endodontic perforation, bioceramic materials, iatrogenic perforations, pathological perforations, apical perforation, and coronal perforation." The search covered the period from 1989 to 2024. The articles were chosen based on the inclusion criteria. The initial search elicited 42 articles, of which 33 were finally included in the review. Ethical approval for the study was obtained from the Ethical Review Committee of Riyadh Elm University.

Inclusion and Exclusion Criteria

We included articles in English that were published from 1989 to 2024, involving patients with root perforation. We excluded articles published in languages other than English, unpublished articles, and articles published before 1989.

Discussion

Perforation may occur due to caries, external and internal resorption, trauma, as well as iatrogenic causes [8]. Many studies have suggested that perforation can be identified by radiographs, unexpected bleeding and pain during instrumentation, blood on paper points, an electronic apex locator, and a dental operating microscope [9-11]. Various materials have been used in endodontic perforation repair, from older materials such as indium foil to, more recently, MTA and other bioceramic materials [12]. These materials must have certain properties, such as biocompatibility, bacteriostatics, and radio-opacity [13]. Of the materials that have demonstrated significant results in perforation repair recently, most articles highlight MTA and bioceramic materials due to their high sealability and several other qualities [14,15].

The techniques applied in the repair of endodontic perforation depend on the condition of the perforation. For example, the orthograde approach is used immediately after the perforation occurs, as compared to orthodontic extrusion in the case of a single-rooted tooth, internal matrix for a large multi-rooted tooth, intentional replantation for large perforation size and when orthograde or surgical approaches cannot be done, and finally the surgical approach in cases of bone resorption or healing failure of previously treated tooth [10,13]. Many studies have shown that the prognosis of the perforated root varies depending on many factors [9,13,16]. These factors include time elapsed after perforation, the location and size of the perforation, the material used for perforation repair, and systemic factors [15].

Etiology

Root perforation can be caused by either iatrogenic or non-iatrogenic reasons [8]. Insufficient knowledge of tooth anatomy and morphology, especially the internal structure, can result in iatrogenic perforation at any stage of root canal treatment. External or internal root resorption and tooth furcation associated with caries are examples of non-iatrogenic perforation [13]. As such, cone-beam CT (CBCT) has become increasingly important for assessing and identifying root perforation [17].

Iatrogenic Perforation Types

Coronal third perforation: Coronal third perforation occurs during the process of canal orifices exploring and enlargement; its incidence increases at wide crown-root angulation, invasive dentine removal at the coronal part, canal misrecognition due to calcification of canal space, and underestimating tooth inclination, especially at anterior teeth [9,18].

Middle third perforation: Middle third perforation appears when there is aggressive instrumentation to narrow or curved canals, which sometimes drift away from the canal center pathway. Furthermore, the intense use of rotary files or ultrasonic units while negotiating a calcified canal increases the incidence of middle third perforation [9].

Apical third perforation: Apical third perforation occurs at the apical site of the root resulting from instrument inclination leading to canal straying away from root midline, which causes zipping or ledge formation. Zipping perforation can result from poor insertion of a rigid endodontic file inside a curved canal. Apical third perforation can also occur when passing the apical constriction strongly during the cleaning and shaping stage of root canal treatment [9].

Perforation in post-space preparation: Preparing the post-space after root canal obturation may lead to perforation. The risk of root perforation increases in cases of poor preparation of canals for post-space without sufficient knowledge of root canal anatomy or inadequate armamentarium, which may lead to significant damage to the surrounding dentine [9].

Pathological Perforation (Non-iatrogenic)

Pathological perforation may occur due to tooth caries or root resorption. Root resorption refers to the gradual loss of dentine and cementum due to the ongoing activity of osteoclastic cells [19]. When root resorption occurs within the root canal system, it is referred to as internal inflammatory root resorption, a condition that appears radiographically as an oval-shaped enlargement of the root canal system. Although the precise cause is idiopathic, the condition may appear in post-pulpotomy treatment, pulpal inflammation, and trauma cases. Internal inflammatory root resorption is rare and typically an isolated event, but it can develop into a perforation; therefore, it is crucial to control the disease with early detection and intervention before it reaches this stage [9,20].

Destruction of the cementum and periodontal ligament cells on the root surface is known as external inflammatory root resorption. External resorption has different types, all of which may progress until the resorptive defect reaches the root canal walls [9]. The type, site, and extent of resorption are all determinant factors for its management [20,21]. Additionally, extended carious lesions may advance to root perforation. Carious lesion is defined as damage to dental tissues caused by microbial action. Carious lesions that have not been controlled may result in either perforation of the pulp chamber floor or an extension within the root, causing root perforation. Root canal treatment, crown lengthening, and root extrusion or root resection to retain sufficient radicular segments may be needed to treat such perforations [9]. However, tooth restorability is unfavorable in most of these cases [22].

Diagnosis

Root perforation must be diagnosed immediately to provide the correct treatment, improve the prognosis, and stop bacterial colonization [23]. Postponing diagnosis and treatment of root perforation may result in further complications and even tooth loss [24]. To establish the diagnosis, specific signs and methods must be used [25]. The location and existence of perforation can be identified via radiographs, unexpected bleeding, discomfort during the instrumentation process, using an electronic apex locater, as well as a dental operating microscope [10].

Radiographs: Radiographic assessment is a crucial aspect in the management of endodontic disorders, from diagnosis and planning the course of treatment to assessing the results [25]. Periapical radiography is the imaging technique that is most often recommended for endodontic diagnosis, treatment planning, and follow-up [15]. The diameter and location of the perforation can be assessed using CBCT, which offers three-dimensional images of the tooth [26].

Bleeding and pain: The classic warning signs of root perforation below periodontal attachment include profuse bleeding into the pulp chamber and sudden pain experienced during instrumentation and post-space preparation. Saliva or irrigating solution leakage into the access cavity occurs when the perforation is coronal or above the periodontal attachment. In addition, patients often complain of the taste of irrigating solutions when perforation occurs [25]. Finally, blood on a paper point can also be a strong indicator of perforation occurrence [15].

Electronic apex locator: The apex locator is a technological device that can help in detecting root perforation [15]. It functions by placing a file onto the canal; if perforation is present, measurement with the device will show a reading of 0, indicating that the periodontal ligament is communicating with oral cavity tissues [9].

Dental operating microscope: The dental microscope is a reliable instrument for perforation detection [25]. It is an excellent choice for visualizing the location and extent of the perforation owing to its bright operating light and high magnification [9].

Methods of Management

Materials used for endodontic perforation repair must meet several criteria. First, biocompatibility is essential to ensure the material does not provoke adverse reactions in the oral cavity or body. Second, antimicrobial properties are necessary to inhibit microorganism proliferation. Third, a robust sealing material must be applied to the perforated root area to ensure a favorable prognosis. Additionally, osteogenic and cementogenic properties are important to prevent bacterial leakage and ongoing bone resorption. Radiopacity is also preferred for clear imaging during treatment and follow-up visits. Finally, the availability of cost-effective materials is important to minimize the financial burden on patients and dental clinics [13].

In the past, different endodontic and restorative materials, including amalgam, glass ionomer cement, zinc oxide-eugenol cement, super 2-ethoxybenzoic acid (super EBA), gutta-percha, composite resin, and Cavit were used for perforation repair. However, as these materials were not biocompatible and had suboptimal outcomes, new materials such as MTA, biodentine, endosequence, and BioAggregate have been explored [27]. Table 1 summarizes the properties of bioceramic materials used for root perforation repair.

**Table 1:** Characteristics of suitable materials used for perforation repair Table created by the authors

Material	Biocompatibility	Sealing ability	Antibacterial	Osteogenesis effect
Mineral trioxide aggregate	✓	✓	✓	✓
Biodentine	✓	✓	✓	✓
Endosequence	✓	✓	✓	✓
BioAggregate	✓	✓	✓	✓

MTA: Recently, MTA was introduced for use as a repairing material in endodontic perforation [12]. It is a bioceramic material composed of calcium silicate, and it is generally used in endodontics [23]. MTA exhibits numerous beneficial properties, such as excellent sealing ability, biocompatibility, antibacterial effects, radiopacity, and capability to set even in the presence of blood [26]. According to a recent systematic review by Borges et al., marginal adaptation of MTA, in comparison with other repairing materials in root-end cavities, has demonstrated perfect conformity to the dentinal walls [15]. MTA has a high success rate, attributed to its ability to effectively seal defects and promote healing through its osteogenic properties, which facilitate the regeneration of cementum and stimulate bone formation [28]. However, the drawbacks of MTA include discoloration, difficulty in removal, extended setting time, and high cost [14].

Biodentine: Biodentine is a new type of calcium silicate-based bioceramic material whose main components are tricalcium and dicalcium silicate, excluding the calcium aluminate, bismuth oxide, and calcium sulfate found in MTA. Despite tricalcium silicate being present in both MTA and biodentine, biodentine features denser particles and less porous structures; consequently, biodentine demonstrates superior mechanical properties, easier manipulation, highly alkaline pH, and a shorter setting time compared to MTA [29,30]. However, the adhesion ability of biodentine is weaker than that of MTA in cases of blood contamination. Interestingly, in the absence of blood contamination, biodentine exhibits superior bonding strength compared to MTA, which some studies have attributed to its smaller particle size [31].

Endosequence: Endosequence, otherwise known as endodontic root repair material (ERRM), mainly consists of zirconium oxide, monobasic calcium phosphate, and tantalum oxide. It is commercially available as a pre-mixed, ready-to-use compound, which ensures material consistency and facilitates clinical application. ERRM is a biocompatible, insoluble, hydrophilic, and bioactive material that does not contain aluminum. The setting of ERRM begins upon exposure to moisture, which is advantageous and provides an excellent sealing property. These attributes make ERRM suitable for the treatment of dental perforation [23]. Some in vitro studies have indicated that ERRM exhibits a sealing ability comparable to that of MTA [26], whereas others have found that the former has a higher sealing ability than the latter [30].

BioAggregate: BioAggregate is a bioceramic cement composed of tricalcium silicate, dicalcium silicate, calcium phosphate monobasic, amorphous silicon dioxide, and tantalum pentoxide. One study suggested that BioAggregate can promote the formation of mineralized tissue and the precipitation of apatite crystals that grow larger over time with increased immersion, hence indicating its bioactivity [30]. A study by Hashem et al. concluded that MTA was more affected by acidic pH conditions than BioAggregate when utilized as a material for perforation repair [12,31].

Techniques for Managing Root Perforation

The goal of managing perforation is to regenerate healthy periodontal tissues at the perforation site without ongoing inflammation or loss of periodontal attachment. In cases where there is periodontal breakdown, the goal of treatment shifts to re-establishing tissue attachment. Therefore, successful perforation repair relies on effectively sealing the perforation and restoring a healthy periodontal ligament. Regardless of the site, size, or time to repair, treatment must be offered to prevent further complications. The two treatment options in such cases are repair or extraction. First, the tooth must be evaluated for restorability. Extensive pathological perforation typically renders the tooth unrestorable. If the tooth is deemed unrestorable or endodontic treatment is impossible to complete, the patient should be informed about the benefits of extraction and potential prosthodontic options. For some teeth, accessing the perforation may pose a significant risk of collateral damage or failure, making extraction the only viable option. If the tooth is deemed restorable, repair may be considered [12].

As good visibility is crucial to properly assess the damaged site, access to an operating microscope is recommended [9]. Timely sealing of the perforation, the type of material used, the location of the perforation, and appropriate sealing of the perforation are all important factors in successful perforation management. There are two types of perforation management approaches: nonsurgical and surgical. Nonsurgical management of perforation includes the orthograde approach, the management of crestal root perforation, and intentional replantation [10].

Orthograde approach: Fresh perforation that occurs during endodontic or operative procedures often results in hemorrhage. Hemorrhage can be controlled first by applying pressure or irrigation, followed by adequate sealing of the perforation. Using hemostatic agents and materials that arrest bleeding such as calcium hydroxide can control bleeding. For example, calcium hydroxide can be syringed into the canal and allowed to remain for 4-5 min before flushing with NaOCl. This technique should be repeated 2-3 times. Other hemostatic materials used to control bleeding include collagen, calcium sulfate, freeze-dried bone, and MTA. Materials used in the orthograde approach can be absorbable barrier materials such as collagen and calcium sulfates, or nonabsorbable barrier materials such as MTA, super EBA, resin cement, composite bonded restoratives, and calcium phosphate cement [10].

Management of crestal root perforation: Biocompatible materials with a short setting time and good sealability properties should be used for sealing. Orthodontic extrusion is recommended for a single-rooted tooth to bring the perforation to a coronal position for external sealing without surgical intervention [10]. For large perforations in the furcal region of molars, the internal matrix technique is suggested to avoid extrusion of the repair material. The best materials for furcation perforation are MTA, iRoot BP, calcium-enriched mixture cement, ProRoot MTA, and biodentine [13].

Intentional replantation: Intentional replantation is considered when neither orthograde nor surgical therapy is possible. The technique is used when a perforation is too large to repair or cannot be repaired without removing too much bone. The tooth should be extracted atraumatically to prevent damage to the surrounding tissues, immediate repair of the perforation must be completed, and replantation should be done promptly. Common complications of this technique include inflammatory root resorption and ankylosis [13].

Surgical approach: In cases of large perforation (e.g., resorption) or failure of healing after nonsurgical repair, a surgical approach is one management option. Parameters to be considered before surgical management include the amount of bone remaining, the extent of osseous destruction, duration of defect, periodontal disease status, attachment level of soft tissue, oral hygiene, and the surgeon’s expertise in tissue management. For visibility of the perforation site, a buccal full-thickness flap is raised following osteotomy to reach the site; then the perforation is sealed with sealing material, and finally, sutures are placed on the flap [13].

Prognosis

Perforation of the root canal system has a serious impact on tooth prognosis owing to the direct communication between the root canal system and the periodontium [6]. Studies have shown that the prognosis of the perforated root depends on many factors [16,9,13], including time elapsed after perforation, location and size of the perforation, the material used for the repair, as well as systemic factors [15].

Time: Time-based prognosis depends on two types of perforations, these types known as fresh perforation and old perforation. fresh perforation occurs during the endodontic procedure and is manifested by fresh blood at the affected site. However, old perforation is the perforation which left untreated during the same time of the dental appointment [10]. The best prognosis occurs when management of the perforation takes place immediately. While the old perforation leads to bacterial infection either from periodontium or by carious lesion, timely management will help to decrease the possibility of spreading of infection to the periodontium and adjacent tissue, providing a more favorable environment for healing [9]. Despite the defect being created in a sterile environment, any delay in sealing the perforation can result in wound infection, thereby hindering the recovery process [17].

Size: Many authors suggest that small perforation size allows for tight sealing of the affected area, leading to a more favorable prognosis [9,10,13].

Location: Success in managing perforation is determined by the presence or absence of bacterial contamination at the perforation site. Perforation near the crestal bone and epithelial attachment are critical due to the increased risk of contamination from the oral environment through the gingival sulcus. Perforation coronal to the crestal bone is more accessible for repair, allowing for restoration without periodontal involvement. Although a favorable prognosis for perforation apical to the crestal bone and epithelial attachment has been noted, the outcome is contingent upon effective cleaning, shaping, and obturation procedures [30].

Material: Regardless of the cause or location of the perforation, the prognosis of root perforation is influenced by the chemical and physical properties of the materials used. Therefore, endodontists should utilize materials that have favorable characteristics [22]. The best materials to be used are biocompatible materials, which have antibacterial action, osteogenesis action, and provide better sealability [14].

Systemic factors: In addition to clinical factors, Holland et al. concluded that the repair process of perforated teeth also depends on systemic factors such as chronic diseases, hormone imbalance, and age, which can affect host immunity and thus the outcome of root canal treatment and healing process [32].

Additional factors: Some other factors such as pulp status, tooth type, and tooth location (maxillary or mandibular) have been reported, but these findings require further investigation to verify their effect [15,33].

**Figure 1:**
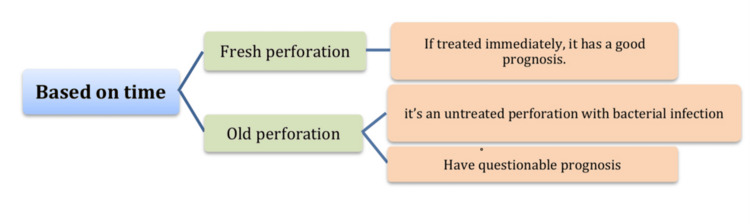
Factors affecting prognosis of root perforation (time) Figure 1 created by authors

**Figure 2:**
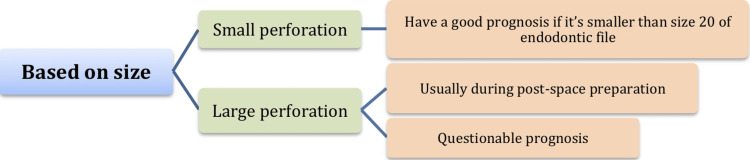
Factors affecting prognosis of root perforation (size) Figure 2 created by authors

**Figure 3:**
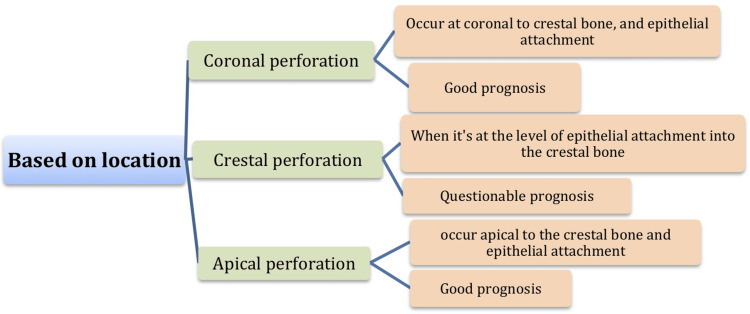
Factors affecting prognosis of root perforation (location) Figure 3 created by authors

Figures 1-3 summarize the main factors affecting the perforated root prognosis.

## Conclusions

Root canal perforation involves communication between the root and external oral tissue, which can occur due to iatrogenic or pathological reasons. A proper diagnosis for root perforation should be done immediately once it is explored with assistive methods. Management of root perforation depends on many factors, such as clinicians' comprehensive knowledge of tooth anatomy, good clinical expertise, and prepared clinical settings such as the availability of advanced armamentarium and sealing materials. Moreover, the prognosis of root perforation depends mainly on the time passed before repair, perforation size, and perforation site. However, some studies have highlighted the impact of systemic factors on root perforation prognosis, and further research needs to be conducted to confirm such an influence.
